# The Influence of Granite Cutting Waste on The Properties of Ultra-High Performance Concrete

**DOI:** 10.3390/ma12040634

**Published:** 2019-02-20

**Authors:** Íñigo López Boadella, Fernando López Gayarre, Jesús Suárez González, José Manuel Gómez-Soberón, Carlos López-Colina Pérez, Miguel Serrano López, Jorge de Brito

**Affiliations:** 1 Campus de Gijón, Department of Construction and Manufacturing Engineering, University of Oviedo, 33203 Asturias, Spain; lopezinigo@uniovi.es (I.L.B.); suarezg@uniovi.es (J.S.G.); lopezpcarlos@uniovi.es (C.L.-C.P.); serrano@uniovi.es (M.S.L.); 2 Department of Architecture Technology, Polytechnic University of Catalonia, Avda Diagonal 649, 08028 Barcelona, Spain; josemanuel.gomez@upc.edu; 3 CERIS, Department of Civil Engineering, Architecture and Georresources, Instituto Superior Técnico, Universidade de Lisboa, Av. Rovisco Pais, 1049-001 Lisbon, Portugal; jb@civil.ist.utl.pt

**Keywords:** ultra high performance concrete, steel fibers, granite cutting waste, recycled concrete

## Abstract

This study analyzes the effect of using waste by-products generated in the process of granite cutting as part of the granular structure of Ultra High Performance Concrete (UHPC). The manufactured concrete has a compressive strength greater than 115 MPa. This study substitutes 35%, 70% and 100% of the volume of micronized quartz powder (<40 μm) with granite cutting waste. This is an innovative study where the feasibility of using waste from granite quarries as a replacement for micronized quartz in UHPC has been analyzed. The results show an improvement in the workability and compressive strength of UHPC, for all substitution ratios. The flexural strength and tensile strength increase when the substitution ratio is 35%, and even the values obtained for 100% substitution are acceptable. In view of the results obtained in this study, granite cutting waste, instead of the micronized quartz powder usually used, is a viable alternative for the manufacture of expectedly more sustainable UHPC.

## 1. Introduction

In recent decades, the overexploitation of natural resources has reached unsustainable levels. Overexploitation depletes resources, destroys natural habitats and pollutes the environment. The hole in the ozone layer and the greenhouse effect are two of the most serious results. For these reasons, the European Union (EU) is enforcing policies to reduce the impact of waste materials on the environment while improving the efficiency of resource management within the common territory. In 1973, the EU launched a corrective policy, called the Environment Action Program, currently in its seventh edition [1].

The cement and concrete industries are among the main sources of greenhouse gas emissions. In order to improve the sustainability of the building sector with more environmentally friendly concrete, studies are being carried out using recycled materials or by-products from the building sector as partial or total replacements of natural aggregates in the manufacture of Ultra High Performance Concrete (UHPC).

Soliman and Tagnit Hamou [2] studied the effect of using glass waste as partial or total substitutes of quartz sand in UHPC. For a 50% ratio, an UHPC with a workability and compressive strength similar to those of the reference concrete can be obtained. Also, an UHPC with a very dense microstructure is achieved. Zegardlo et al. [3] and Gonzalez-Corominas and Etxeberria [4] analyzed the influence of using ceramic recycled aggregates on the properties of UHPC and high-performance concrete (HPC). They found an increase in the compression strength of 24% and an increase in the tensile strength of 34% of the UHPC, when 100% of sand was replaced with recycled ceramic waste, and improved properties of the HPC up to 30% replacement of fine aggregate with ceramic waste.

Al-Jabri et al. [5] and Ambily et al. [6], studied the use of copper slag as substitute of fine aggregates, while Zhu et al. [7] used iron ore tailings. The results obtained in these studies show a promising future for the use of industrial by-products in the manufacture of HPC or UHPC. On the other hand, the results obtained by Al-Jabri et al. [5] showed an improvement in the properties of HPC when the content of copper slag increased. For a ratio of 100%, there are increases of 22% in compressive strength, 27% in flexural strength and 19% in tensile strength.

UHPC is a promising building material with great compressive strength and excellent behavior against flexural stresses when short steel fibres are added. The Association Française de Génie Civil differentiates them from HPC, since they have a compressive strength higher than 150 MPa, although this limit is only indicative and when failure occurs it is not brittle due to the presence of different types of fibre [8]. Generally, concrete with a compressive strength higher than 100 MPa can be considered UHPC. Orgass and Klug [9] observed that when the content of fibres increases, the flexural strength of UHPC increases proportionally. In addition, ductile post-cracking behavior was observed. The results of Wu et al. [10] are in the same line. With an addition of the 3% steel fibres, they achieved a compressive strength above 150 MPa and a flexural strength greater than 35 MPa. Park et al. [11] reported an improvement in the post-cracking flexural properties of UHPC with an increase of the fibres length.

However, it is not an ecologically sustainable material as it requires high cement contents, resulting in high energy consumption. Accordingly, the development of more sustainable concrete, incorporating materials that require lower energy consumption in the manufacturing process, is currently being exploited. This can be achieved by reusing industrial by-products. In this regard, Pyo and Kim [12] studied the feasibility of incorporating different types of industrial by-products in UHPC. The results showed that it is possible to replace micronized quartz with coal bottom ash and fly ash with no significant loss of workability or of compressive strength. By using blast furnace slag, as a partial substitute of cement or silica fume, the UHPC workability is improved without affecting its compressive strength. Pyo et al. [13] studied the feasibility of using fly ash or blast furnace slag as partial substitutes of cement. They observed that the mechanical properties of UHPC, with a substitution ratio of 30% of blast furnace slag, were similar to those of the reference concrete. However, when they used fly ash, a decrease in the mechanical properties of UHPC was observed. In this line, Randl el al. [14] obtained compressive strength similar to that of the reference concrete for a 45% replacement ratio of cement with extra fine blast furnace slag. The use of a 45% of fly ash caused a loss of 25%.

Following the same line of research, Ghafari et al. [15] evaluated the influence of the replacement of silica fume with fly ash or blast furnace slag on autogenous shrinkage. The results showed that for 50% and 100% ratios the autogenous shrinkage falls due to a decrease in the number of fine pores. Yazici et al. [16] investigated the effect of fly ash and blast furnace slag on the compressive strength of reactive powder concrete cured in autoclave. The results show that, by using fly ash and/or blast furnace slag, the content of cement and silica fume can be reduced. In addition, by reducing the content of silica fume, the superplasticizer content is considerably reduced.

Edwin et al. [17] investigated the use of copper slag in contents of 5%, 10%, 15% and 20% of cement weight. The results show an improvement of the mortar’s workability as the copper slag content increases. In terms of compressive strength at 90 days, the results are comparable or even better than those of the reference concrete. Also, with the replacement of cement with copper slag, the hydration of the cement pastes can be slowed.

The use of micronized quartz (crystalline silica) in the manufacture of UHPC reduces the cement content and completes the size grading curve in the smallest sizes, thanks to the size of its particles (<40 μm). The packing density of the matrix is thus increased, achieving greater concrete compactness, stability and durability. However, the utilization of micronized quartz, (not unlike the use of silica fume), has a high energy cost and increases the CO_2_ footprint due to the grinding process. It can also cause serious health problems, such as silicosis [18]. Various studies have been carried out to find alternative materials. For example, Burroughs et al. [19] analyzed the effect of substituting silica powder with limestone filler. Although a loss of strength was observed, it was less than 6% for a substitution ratio of 100%. Soliman and Tagnit Hamou [20] managed to improve the workability of UHPC and increase its compressive strength when 100% of micronized quartz was replaced with glass powder. This is due to an improvement in the packing density and to the high strength and modulus of elasticity of the glass particles. The results of Vaitkevicius et al. [21] are in the same line. For a replacement ratio of 100% of micronized quartz, they achieved a compressive strength of 221 MPa, compared to 182 MPa of the reference concrete.

For all these reasons, and because the mining industry sector is second only to the building sector in the amount of waste it generates [22], this study explores the viability of using waste from a granite quarry as a partial or total substitute of micronized quartz to produce expectedly more sustainable UHPC. It is an innovative study since so far this type of waste has not been used in the manufacture of UHPC.

## 2. Experimental Study

### 2.1. Materials

**Figure 1:**
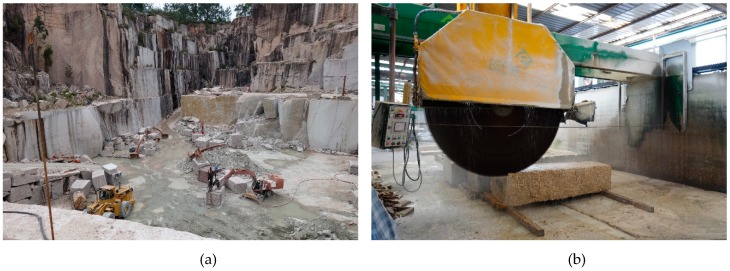
Granite powder waste production. (**a**) Granite quarry; (**b**) process of cutting granite blocks.

**Figure 2:**
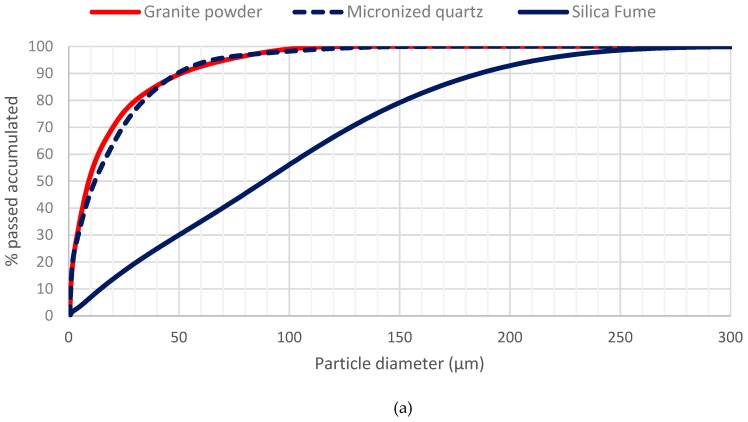

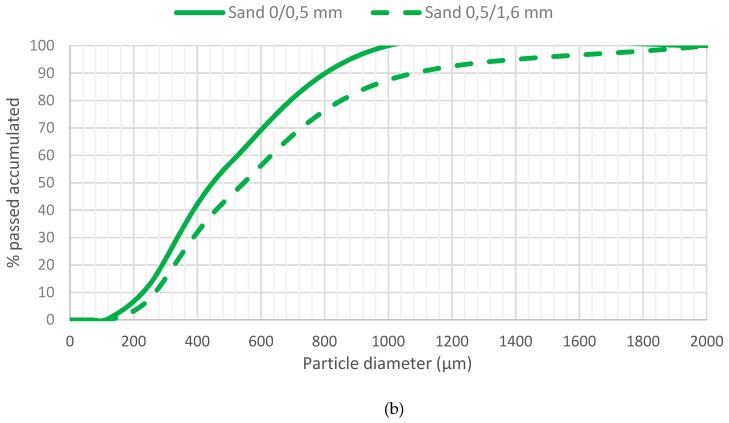
Size distribution. (**a**) Granite powder, micronized quartz and silica fume; (**b**) coarse and fine sand.

The cement used was CEM I 42.5 R/SR. Table 1 summarizes its main features. Two silica sands with size fractions of 0/0.5 mm and 0.5/1.6 mm were used. As additions, densified silica fume, with an average particle size of 0.15 μm and micronized quartz powder with an average particle size of 20 μm were used. To achieve optimal workability, two types of polycarboxylate superplasticizers were used. The superplasticizers used were ViscoCrete-225 Powder (Sika, Madrid, Spain) and Basf MasterEase 5025 (Basf, Barcelona, Spain). The short steel fibres used in this study had a diameter of 0.2 mm and a length of 13 mm. Finally, granite powder waste (FG), with an average particle size of 20 μm, obtained from the cutting process of granite blocks was used as a supplementary material (Figure 1a,b). Table 2 summarizes the properties of the materials used. The densities shown there were determined using a helium pycnometer. Figure 2a,b highlight the differences in size between powder and sands and show the size distribution of the powder materials. The chemical composition of micronized quartz and FG was determined by X-ray fluorescence analysis. Table 3 shows the corresponding results.

### 2.2. UHPC Design

To carry out this study, a reference mix was designed that ensures a self-compacting fresh concrete with a compressive strength above 115 MPa. Once the characteristics of the reference concrete were verified, 35%, 70% and 100% of the micronized quartz was replaced by the same volume of granite powder. Table 4 shows the composition of each manufactured UHPC, in kg/m^3^.

### 2.3. Experimental Program

The experimental program includes four mixes. The first one is the reference UHPC. The rest of the mixes correspond to the UHPC with different contents of granite waste (Table 4).

The manufacturing procedure was as follows: First, the two silica sands were introduced into the mixer. Next, micronized quartz or the granite powder was added, then silica fume, and finally cement. Once all the dry materials were in the mixer, they were mixed for 30 s before the water was added. Mixing continued for two more minutes, after which the superplasticizer was added and, finally, the steel fibres. The final mixing lasted 25 min.

12 specimens of each of the five mixes were manufactured according to the UNE-EN 12390-1 standard [23]: Three 15 cm × 30 cm cylindrical specimens, three 10 cm × 10 cm × 10 cm cubic specimens and three 10 cm × 10 cm × 4 0 cm prismatic specimens. The specimens were cured for 28 days as specified in UNE-EN 12390-2 [24].

The consistency of fresh UHPC [25], its density [26], modulus of elasticity [27], compression strength [28], flexural strength [25] and tensile strength [25] were determined. To determine consistency, a truncated cone mould with 200 mm and 130 mm in diameter, and 200 mm in height was used. These sizes are slightly larger than those recommended in NF P18-470. The modulus of elasticity, compressive strength, flexural strength and tensile strength were calculated after the 28-day curing process in a wet chamber with a temperature between 20 ± 2 °C and a relative humidity higher than 95%. These calculations are described in detail in the following section.

## 3. Analysis of the Results

### 3.1. Workability

**Figure 3:**
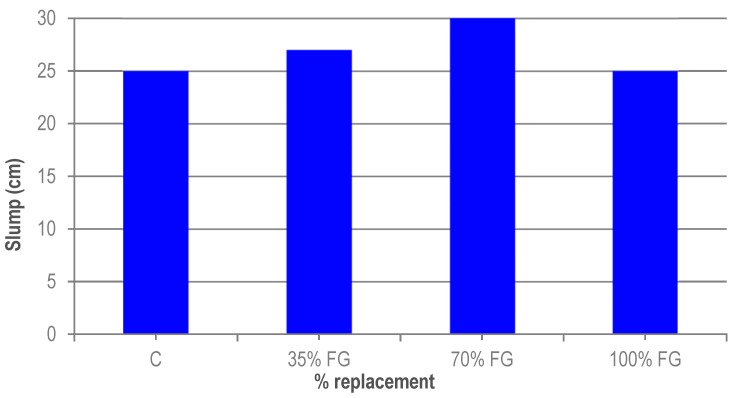
Slump of UHPC.

The workability of UHPC was determined after the mixing process. Figure 3 shows an increase in the workability of UHPC when granite waste replaces micronized quartz. Also, the workability improves as the replacement ratio increases, up to a point. The highest value is obtained for the 70% ratio. The mixes with a 100% replacement of micronized quartz with granite waste had a slump value equal to that of the reference concrete. This may be due to the more rounded shape of the granite cutting waste particles, although when the substitution ratio is 100% the compactness of UHPC falls. This increase in the workability of UHPC is in line with the results obtained by Soliman [20,29], where the incorporation of glass powder as a substitute for micronized quartz or silica fume improves the workability of UHPC.

### 3.2. Density of the Hardened UHPC

**Figure 4:**
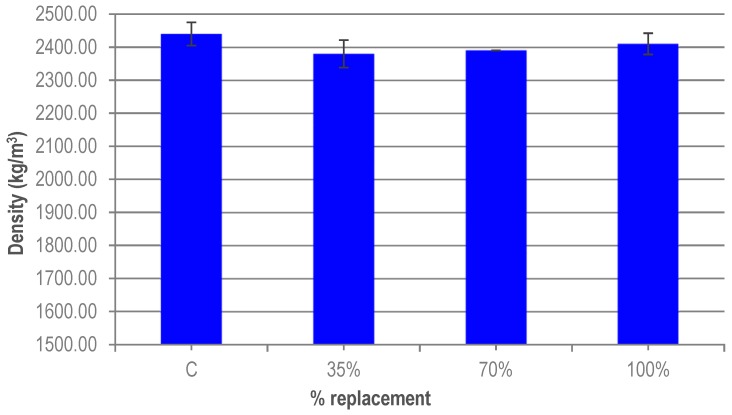
Density of UHPC.

The density of the hardened UHPC was measured after 28 days. Figure 4 shows the values obtained for the different replacement ratios. A slight reduction in the density of the UHPC with granite waste is observed relative to the reference UHPC, although the values are very similar. This may be due to the smaller specific surface of the granite powder particles, increasing the effective water and the slump and, therefore, the density of the UHPC is slightly smaller than that of the reference concrete. The variation between the 35% and 100% replacements is less than 1.5%. These variations can be due to the variability in the results obtained, as seen in Figure 4 (error bars). Summing up, the substitution ratio of the granite cutting waste has no impact on the density of UHPC.

### 3.3. Compressive Strength

**Figure 5:**
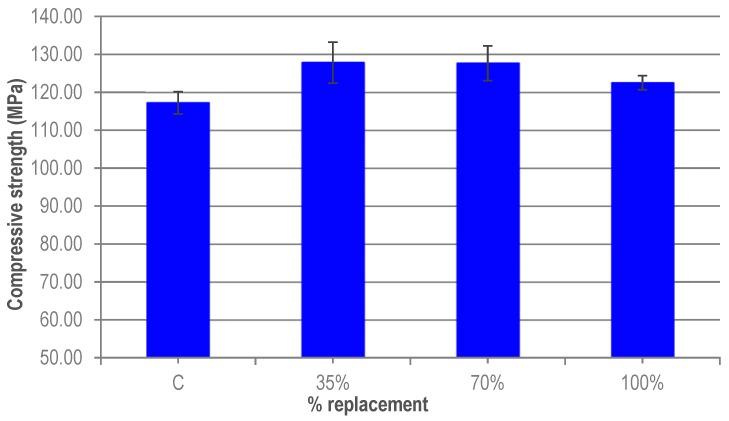
Compressive strength of UHPC.

The results shown in Figure 5 correspond to the average strength from a series of three specimens corresponding to each of the different replacement ratios. There is an increase in the average compressive strength in all the mixes with granite waste. This increase in compressive strength oscillates between 8.5%, for ratios of 35% and 70% and 4.5% for 100%. These slight increases can be due to the better compactness of the mixes when the granite cutting waste is incorporated. Again, the results (35%, 70% and 100%) are within the expected error of the test and, although the compressive strength is slightly higher, the substitution ratios have no great influence on the compressive strength. Since the hardness of micronized quartz and granite waste is similar, the increase in the compressive strength may be due to a greater compactness of the concrete, as the mixes with granite waste have greater fluidity than the reference UHPC. It may also be due to the greater number of finer particles present in the granite waste.

These slight increases, in the compressive strength of UHPC are in agreement with the results of Soliman [20] and Vaitkevicius [21]. In the first case, replacing 100% micronized quartz with glass powder produced an increase in long-term resistance greater than 12%. Vaitkevicius [21] observed that the substitution of 100% of micronized quartz with glass powder increased the compressive strength by more than 20%.

### 3.4. Modulus of Elasticity

**Figure 6:**
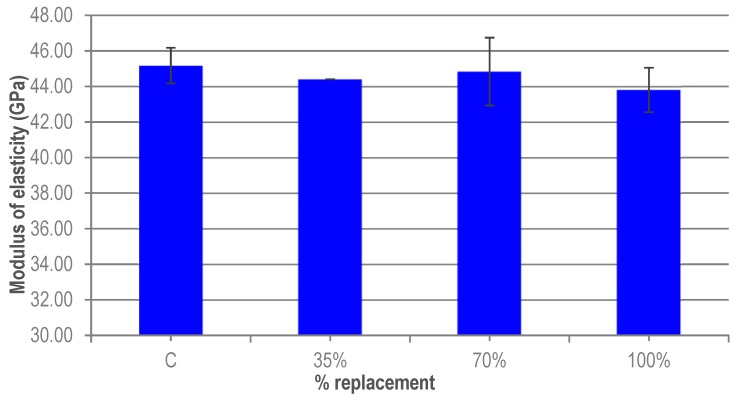
Modulus of elasticity of UHPC.

The tests to determine the modulus of elasticity were carried out after 28 days. Three 15 cm × 30 cm cylindrical specimens were used per replacement level. Figure 6 shows the influence of granite waste on the modulus of elasticity of UHPC. First, a reduction in the modulus of elasticity of the UHPC with granite waste is observed with respect to the average value obtained for the reference UHPC. However, the variation that is produced is very small, less than 5%. The results obtained were not expected. They can be attributed to the fact that the variability of the results predominates over the variability of the variable analyzed (% substitution).

Regarding this, variations between the results obtained in different studies have been observed. The results of Yazıcı [30] show a reduction of the modulus of elasticity of UHPC when incorporating fly ash or blast furnace slag. This decrease is attributed to a loss of stiffness. However, the studies of Pyo et al. [12] and Safiuddin et al. [31] show an increase in the modulus of elasticity when different types of industrial by-products were incorporated. Safiuddin et al. [31] attributed this increase in modulus of elasticity to a micro-filler effect and a reduction in the porosity of HPC produced by the industrial by-products.

### 3.5. Flexural Strength

**Figure 7:**
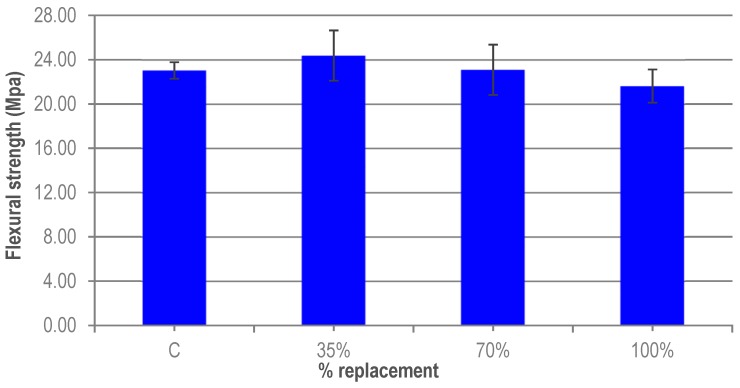
Flexural strength of UHPC.

**Figure 8:**
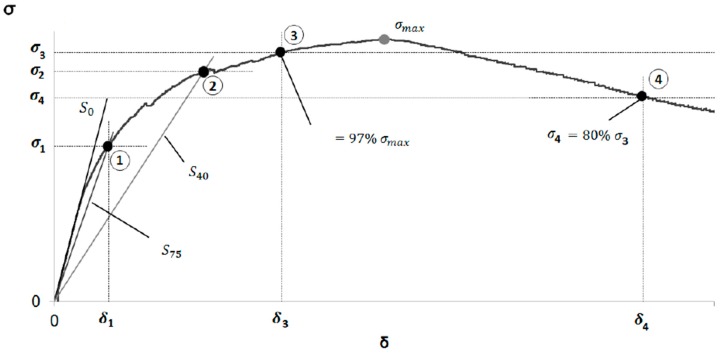
Stress–strain curves and key points.

The results shown in Figure 7 correspond to the average of the results from a series of three specimens manufactured per replacement ratio. The scatter in the results can be due to the random distribution, probably non-uniform, of the fibres in the mix. The average flexural strength is gradually reduced as the granite waste content in the UHPC increases. For the 35% ratio there is an increase in flexural strength of 6% relative to the reference UHPC. This increase is possibly due to a better adherence between the granite waste and the cement paste, as a consequence of the irregular shape of the granite particles. However, for the other ratios, there is a loss of flexural strength. This may be due to an increase in the amount of effective water as the percentage of substitution increases because the specific surface of the FG is less than that of the micronized quartz. When the replacement ratio is 70%, the flexural strength is practically equal to that of the reference UHPC. In any case, the variations are small and due to the variability of the experimental results. All of them fall within the expected error of the test, as seen in Figure 8. The results of flexural strength show a similar behaviour to those obtained by Wu et al. [32].

### 3.6. Tensile Strength

The parameters necessary to determine the tensile strength of UHPC resulted from the stress-strain curves (Figure 8), obtained when performing the flexural tests for each substitution ratio. The key points of stress-strain curves are the following [33].

P_1_ is defined as the intersection between straight S_75_, with a slope equal to 0.75 of the slope of the elastic zone of the stress-strain curve passing through the origin, and the stress-strain curve. P_1_ is defined by parameters δ_75_ and σ_75_. P_2_ is defined as the intersection between straight S_40_, with a slope equal to 0.40 of the slope of the elastic zone of the stress-strain curve passing through the origin, and the stress-strain curve. P_2_ is defined by parameters δ_40_ and σ_40_. P_3_ is defined as the point of the ascending branch at which the equivalent stress is 97% of the maximum stress. P_3_ is defined by parameters δ_loc_ and σ_loc_.

Once the key points of the stress-strain curve are defined, the model of tensile behaviour of the concrete is determined.

E=2.40 h m

$$f_{t}=\frac{\sigma_{75}}{1.63}{\left(\frac{\sigma_{75}}{\sigma_{40}}\right)}^{0.19}$$

$$\varepsilon_{t,u}=\frac{f_{t}}{E}\left(7.65\;\frac{\delta_{loc}}{\delta_{75}}-10.53\right)$$

$$\varepsilon_{t,\;el}=f_{t}/E$$

$$\alpha =\varepsilon_{t,u}/\varepsilon_{t,el}$$

$$f_{t,u}=\alpha^{-0.18}\left(2.46\;\frac{\sigma_{loc}}{\sigma_{75}}-1.76\right)f_{t},$$

where m is the slope of the elastic region of the stress–strain curve; *h* is the thickness of the specimen, in mm; *E* is the modulus of elasticity; *f_t_* is the crack resistance of the matrix reinforced with fibres; *f_t,u_* is the ultimate tensile strength; *ε_t,u_* is the peak deformation

**Figure 9:**
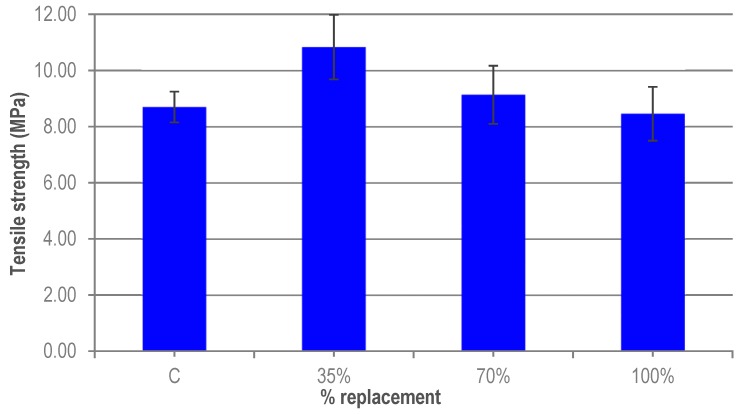
Tensile strength of UHPC.

Figure 9 shows the results obtained. Again, a gradual decrease in strength can be seen when the percentage of granite waste increases. So, for a 35% ratio, there is an increase in the tensile strength of 24%, while for 70% it is only 5%. However, for 100% ratio, the results obtained are similar to that of the reference UHPC. As the error bars show, the high value for 35% may be due to the variability of the experimental results. These good results obtained for the UHPC with granite waste may be due to the better adhesion with the cement paste, as a consequence of the more irregular shape of the granite particles and the presence of the short steel fibres. Also, because these values have been obtained indirectly, through the flexural test the same trend can be seen here. The variation seen for the different levels of substitution may be due to the random nature of the distribution of the fibres in the UHPC.

These results are in line with those obtained by Aldahdooh et al. [34], who used palm oil fuel ash as a partial substitute of cement. The results show an increase in strength up to 50% ratio, when a significant loss occurs. However, a loss of tensile strength when palm oil fuel ash replaces silica fume can be seen. These results are attributed to the higher SiO_2_ content in silica fume compared to cement and palm oil fuel ash.

**Figure 10:**
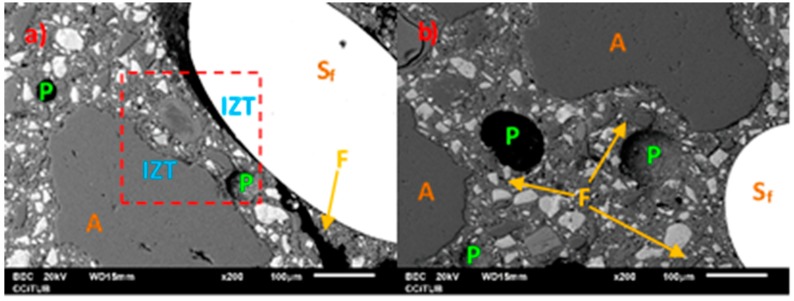

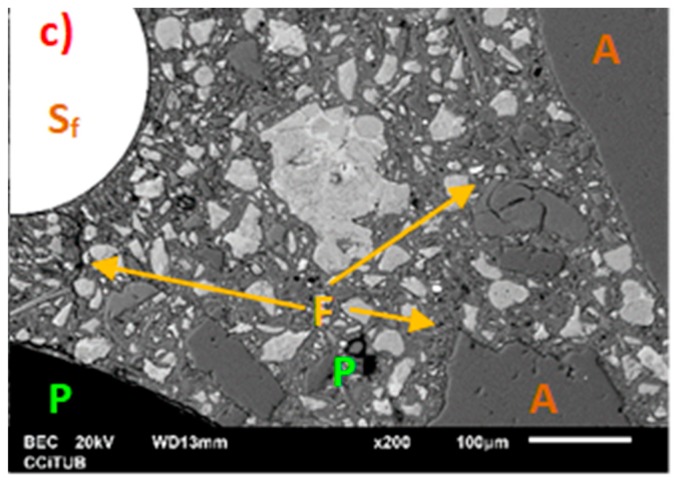
SEM-BEC images of reference concrete (**a**) with 70% (**b**) and 100% (**c**) granite waste; S_f_ Steel fibre, P–pore, A–Aggregate, F–Fissure.

The previous sections indicate that the replacement of micronized quartz with granite waste improves the mechanical behavior, especially the compressive strength. This is probably due to physical issues related to an improvement in compactness due to better distribution of particle sizes. To validate this hypothesis, observations of the microstructure of the samples were made by SEM. It can be observed that in the reference UHPC (Figure 10a), cracks of significant thickness and size are identified, as well as a large interface transition zone (ITZ) that runs in the contour of the steel fibres. Finally, the identified pores have a diameter between 30 μm and 55 μm. However, when the granite waste content reaches 70%, (Figure 10b) and 100% (Figure 10c), micro-cracks that run in the mortar paste are identified. The ITZ has a reduced thickness, it is continuing and follows the roughness of the profile of the surrounding element, both of the aggregate and of the fibre. As for the pores, they increase in number and size.

## 4. Conclusions

Ultra-high-performance concrete (UHPC) has been manufactured using granite cutting waste and the results obtained open the possibility of their use as partial substitutes of the granular skeleton of the concrete.

In some cases, the properties suffer slight increases relative to the reference concrete and in others the variation is so small than the waste incorporation does not influence the analyzed property. The most important conclusions are summarized next.

The workability of UHPC increases as the granite powder waste content is increased. This improvement in the workability of concrete may be due to the lower demand for water by the granite fines, which increases the effective amount of water.

The incorporation of granite cutting waste has no impact on the density of UHPC. The variations are very small, and they are due to the variability of the experimental results.

The greater workability of UHPC as the granite fines content increases favours the compactness of concrete and increases the compressive strength of mixes with granite powder waste. The highest compressive strength is obtained in the 35-70% ratio, with an increment of 9%, although between 3% to 4% can be due to the scatter as result of the random distribution of the fibres.

Regarding the modulus of elasticity, although the results were not as expected, the variability of the results fall within of the expected error and again this property is not clearly affected by the substitution ratio.

## Figures and Tables

**Table 1:** Cement properties.

CEM I 42.5 R-SR	
L.O.I.	≤5.0%
Insoluble residue	≤5.0%
Sulphates	≤3.5%
Chlorides	≤0.10%
2-day compressive strength	20.0 MPa
28-day compressive strength	≥42.5 MPa
	≥62.5 MPa
Initial setting	≥45 min
Le Chatelier expansion	10 mm

**Table 2:** Materials properties.

Property	Silica Fume	Micronized Quartz	Granite Powder	Sand 0/0.5 mm	Sand 0.5/1.6 mm
Density (kg/m^3^)	2300	2609	2633	2616	2616
Absorption at 24 h (%)	-	-	-	0.28	0.53
Sand equivalent, SE (10)	-	-	-	97	97
Humidity (%)	<3.00	<0.20	0.00	0.00	0.00

**Table 3:** Chemical composition (%) of micronized quartz and granite powder.

Specimen	SiO_2_	Al_2_O_3_	Fe_2_O_3_	MnO	MgO	CaO	Na_2_O	K_2_O	TiO_2_	P_2_O_5_	L.O.I
Micronized quartz	>99.3	0.26	0.05	-	-	0.02	-	0.04	0.05	-	-
Granite powder	76.33	11.87	2.00	0.02	0.21	0.43	2.95	5.05	0.13	0.02	0.77

**Table 4:** UHPC composition (kg/m^3^).

Material	Reference	35% FG	70% FG	100% FG
Cement	800	800	800	800
Sand 0/0.5	302	302	302	302
Sand 0.5/1.6	565	565	565	565
Micronized quartz	225	146	68	-
Silica fume	175	175	175	175
Granite powder	-	79	158	225
Water	175	175	175	175
Superplasticizer	10	10	10	10
Steel fibres	160	160	160	160
